# MUREN: a robust and multi-reference approach of RNA-seq transcript normalization

**DOI:** 10.1186/s12859-021-04288-0

**Published:** 2021-07-28

**Authors:** Yance Feng, Lei M. Li

**Affiliations:** 1 National Center of Mathematics and Interdisciplinary Sciences, Academy of Mathematics and Systems Science, Chinese Academy of Sciences, Beijing, China; 2 University of Chinese Academy of Sciences, Beijing, China; 3 Center for Excellence in Animal Evolution and Genetics, Chinese Academy of Sciences, Kunming, China

**Keywords:** RNA-seq, Normalization, Asymmetrically regulated transcription profiles (ART), Skewness, Mode, Multi-reference

## Abstract

**Background:**

Normalization of RNA-seq data aims at identifying biological expression differentiation between samples by removing the effects of unwanted confounding factors. Explicitly or implicitly, the justification of normalization requires a set of housekeeping genes. However, the existence of housekeeping genes common for a very large collection of samples, especially under a wide range of conditions, is questionable.

**Results:**

We propose to carry out pairwise normalization with respect to multiple references, selected from representative samples. Then the pairwise intermediates are integrated based on a linear model that adjusts the reference effects. Motivated by the notion of housekeeping genes and their statistical counterparts, we adopt the robust least trimmed squares regression in pairwise normalization. The proposed method (MUREN) is compared with other existing tools on some standard data sets. The goodness of normalization emphasizes on preserving possible asymmetric differentiation, whose biological significance is exemplified by a single cell data of cell cycle. MUREN is implemented as an R package. The code under license GPL-3 is available on the github platform: *github.com/hippo-yf/MUREN* and on the conda platform: *anaconda.org/hippo-yf/r-muren*.

**Conclusions:**

MUREN performs the RNA-seq normalization using a two-step statistical regression induced from a general principle. We propose that the densities of pairwise differentiations are used to evaluate the goodness of normalization. MUREN adjusts the mode of differentiation toward zero while preserving the skewness due to biological asymmetric differentiation. Moreover, by robustly integrating pre-normalized counts with respect to multiple references, MUREN is immune to individual outlier samples.

**Supplementary Information:**

The online version contains supplementary material available at 10.1186/s12859-021-04288-0.

## Background

The RNA sequencing (RNA-seq) technology has been the primary mean to explore the transcriptome in the past decade. Like the microarray technique, it can profile mRNA and non-coding RNA [1] transcripts with or without strand-specificity [2]. The flexibility of this technique makes it particularly valuable for identification of novel alternative splicing-isoforms [3], assembly of transcriptome [4], and transcript fusion detection [5].

Accuracy is key to the transcript quantification. Despite that RNA-seq avoids the biases due to dye effects and hybridization in the microarray technology [6, 7], other systematic biases such as sequencing depths, transcript lengths, GC-contents, RNA degradation along with variations in RNA isolation, purification, reverse transcription, cDNA amplification, and sequencing have been reported [8–10]. Thus, it is necessary to normalize read counts preceding the downstream quantitative analysis.

One of the most widely used normalization methods is Reads per Kilobase per Million mapped reads (RPKM), [7] and its paired-end counterpart Fragments per Kilobase per Million mapped reads (FPKM), [4]. They assume the total contents of RNA nucleotides remain unchanged across different samples. In RPKM/FPKM, numbers of nucleotides are converted into numbers of transcripts by adjusting transcript lengths. This step is skipped in Counts Per Million (CPM). Similar to RPKM/FPKM, Transcripts Per Million (TPM), [11] assumes the total numbers of transcripts rather than nucleotides remain unchanged across different samples.

The assumption of the constant total RNA contents or transcripts is unrealistic in some situations [12]. Some scaling methods instead estimate the scaling factors according to different criteria. Relative Log Expression (RLE), used in the package DESeq2 [13], estimates the scaling factor as the median ratio of each sample to the pseudo sample of pre-calculated median library. Trimmed Means of *M*-values (TMM), [14]), used in the package edgeR [12, 15], estimates the ratio of RNA production using a weighted trimmed mean of the log expression ratios.

Other methods have their own assumptions. Quantile method [16], widely used to normalize array data, assumes the transcript abundances follow an identical distribution across different samples. The idea is implemented in the packages limma [17]. A more sophisticated method Remove Unwanted Variation (RUV), [18]) utilizes factor analysis of control genes or samples to adjust for the nuisance of technical effects.

Biologists prefer housekeeping genes [19] in normalizing expression profiles. However, the definition of housekeeping genes is debatable, especially for non-model organisms.

The invariant gene set is a statistical counterpart to the housekeeping gene set [20]. In the microarray setting, the invariant set of probes are selected so that within-subset rank difference in the two arrays is small. When there are multiple samples, the invariant gene set are taken as the intersection of all sample pairs. As the size of samples increases, the invariant gene set would be reduced, and possibly close to null. By the same token, the existence of housekeeping genes for a large collection of samples, especially under a wide range of conditions, is questionable. However, in such a situation, either housekeeping genes or an invariant set between a pair of samples can still be defined. This is a key motivation of the multi-reference normalization method proposed in this report.

**Fig. 1:**
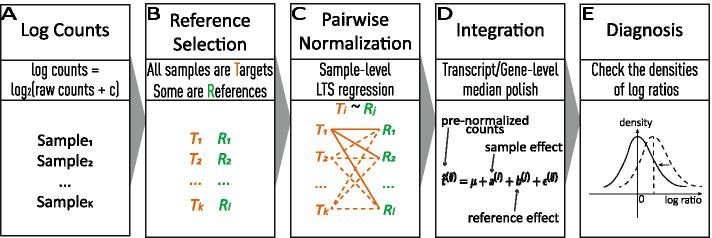
The workflow of MUREN. **A** The workflow takes the raw count table as input followed by log transformation; **B** Take all samples as targets and select a subset of samples as references, see the paragraph "Selection of multiple reference samples" for options; **C** Normalize each target sample with respect to each reference sample; **D** Integrate multiple pre-normalized counts into the final one; **E** Assess the goodness by examine the densities of pairwise log ratios and their skewness before and after normalization

The idea of normalizing pairwise samples with respect to multi-references followed by integrating them via removing the reference effects was initially proposed in the microarray setting [21]. We found the same principle is applicable for RNA-seq data, and proposed two specific parametric models in this report. As illustrated in Fig. 1, we first normalize each pair of target and reference samples by the least trimmed squares (LTS) regression, and then integrate multiple pre-normalized counts by the median polish method to get the final normalized counts. This multi-reference normalizer is implemented as MUREN in R. MUREN is the first approach that carries out pairwise normalization with respect to multi-references in the quantification of RNA-seq transcripts by far.

Crucial for normalization is the evaluation of its goodness. We claim that the goodness includes not only the reduction of bias and variation, but also the preservation of skewness of expression differentiation. The claim is supported by our biological interpretation and statistical analysis of expression skewness, which is exemplified by a single cell data of cell cycle.

## Methods

We propose a two-step normalization procedure for RNA-seq data: pairwise normalization and integration. The introduction of the reference factor allows us to carry out robust normalization with respect to multiple references. The method emphasizes on robustness by adopting least trimmed squares (LTS) and least absolute deviations (LAD) in the two steps respectively. The general scheme of the proposed normalization method is shown in Fig. 1. We start off with a statistical principle of normalization.

### A general statistical model of normalization

Suppose we have two RNA sequencing samples: one reference and one target. Denote the read counts of each transcript indexed by $i$ from the target and reference samples by $\left({T}_{i}, {R}_{i}\right)$ and the true abundances of corresponding transcripts by $\left({\tilde{T }}_{i}, {\tilde{R }}_{i}\right)$ respectively. Ideally, we expect $\left({T}_{i}, {R}_{i}\right)\propto \left({\tilde{T }}_{i}, {\tilde{R }}_{i}\right)$. However, the proportional relationship might be disturbed in the steps of tissue isolation, PCR amplification, and sequencing. The effects of these uncontrollable factors are confounded with true expression level and we need a normalization procedure to adjust the observed read counts. In what follows, we describe a general model for the normalization of RNA-seq data.

Consider a system with $\left({\tilde{T }}_{i}, {\tilde{R }}_{i}\right)$ as input and $\left({T}_{i}, {R}_{i}\right)$ as output. Let $s(\cdot )=\left({s}_{1}(\cdot ),{s}_{2}(\cdot )\right)$ be the system function that accounts for all biases and variations due to uncontrolled biological and technical factors; namely,

1 $$\left\{\begin{array}{c}{T}_{i}={s}_{1}\left({\tilde{T }}_{i}\right)\\ {R}_{i}={s}_{2}\left({\tilde{R }}_{i}\right)\end{array}.\right.$$

Our goal is to reconstruct the input $\left({\tilde{T }}_{i}, {\tilde{R }}_{i}\right)$ based on output $\left({T}_{i}, {R}_{i}\right)$. The model thus describes a blind inversion problem, in which both system $s(\cdot )$ and input $\left({\tilde{T }}_{i}, {\tilde{R }}_{i}\right)$ are unknown.

The blind inversion scheme [22] leads us to think about the underlying relationship between target and reference. As a heuristic start, let us assume that the target and reference sample are biologically undifferentiated. In other word, the differences between target and reference are purely caused by random variations. Statistically, one can assume that the random variables $\left\{\left({\tilde{T }}_{i}, {\tilde{R }}_{i}\right),i=\mathrm{1,2},\dots ,n\right\}$ are independent samples from a joint distribution $\stackrel{\sim }{\Psi }$ whose density centers around the straight line $\tilde{T }=\tilde{R }$, namely,

#### **Assumption R1**

$E\left(\tilde{R }|\tilde{T }\right)=\tilde{T }.$ In this case, ${s}_{1}(\cdot )$ and ${s}_{2}(\cdot )$ are roughly equal to the identity function. Next, we consider the general case. Since only the component of ${s}_{1}(\cdot )$ relative to ${s}_{2}(\cdot )$ is estimable in the pairwise normalization. Thus, we first let ${s}_{2}(\cdot )$ that links the true and observed reference be an identity function, and thus $R={s}_{2}\left(\tilde{R }\right)=\tilde{R }$. In MUREN, we estimate ${s}_{1}(\cdot )$ in the pairwise normalization.

Without loss of generality, we further assume that.

#### **Assumption M**

${s}_{1}(\cdot )$ *is a monotone (increasing) function*.

Then Assumption R1 becomes

#### **Assumption R2**

$$E\left(\tilde{R }|\tilde{T }\right)=\tilde{T }$$, namely, $$E\left(R|g\left(T\right)\right)=g\left(T\right)$$, where $$g(\cdot )= {s}_{1}^{-1}(\cdot )$$.

The next minimization result is the mathematical basis for the regression-based normalization.

#### **Proposition 1**

*Suppose Assumption R2 is valid for some function* $g(\cdot )$. *Then it is the minimizer of* ${\mathrm{min}}_{l}E{\left[R-l\left(T\right)\right]}^{2}$, *which equals* $E\left[R|T\right]$.

This proposition motivates us to estimate g by minimizing the sum of squares

$$\sum_{i=1}^{n}{\left[{r}_{i}-g\left({t}_{i}\right)\right]}^{2}.$$

Finally, we consider the more practical situations. Suppose a portion $1-\lambda (<0.5)$ of transcripts are differentially expressed (DE) by a sufficiently large amount. Then the undifferentiated transcripts can serve as the invariant set of genes for the pairwise normalization, and denote their indices by $U$. Now Assumption R2 is replaced by

#### **Assumption R3**

$E\left( {R_{i} {|}g\left( {T_{i} } \right)} \right) = g\left( {T_{i} } \right),\;{\text{for}}\;i \in U.$Then we estimate *g* by minimizing

$$\sum_{i\in U}{\left[{r}_{i}-g\left({t}_{i}\right)\right]}^{2}.$$

Since $U$ is unknown, we use least trimmed squares (LTS) to minimize the trimmed sum of squares, in the meantime, capture the set of undifferentiated transcripts. Because LTS removes the transcripts with large residuals, which usually are DE transcripts, the correspondence justifies the estimates of LTS.

### Parametrization

We parameterize $g\left(t\right)$ by a simple linear function $\alpha +\beta t$. Consider the regression model

2 $${r}_{i}=\alpha +\beta {t}_{i}+{\varepsilon }_{i},$$

where ${r}_{i}={\mathrm{log}}_{2}\left({R}_{i}+1\right)$ and ${t}_{i}={\mathrm{log}}_{2}\left({T}_{i}+1\right)$ are the log counts. The logarithmic transformation plays the role of variance stabilization to meet the assumption of homoscedasticity in the regression models.

The normalized abundance/count of ${T}_{i}$ with respect to the given reference is then  ${\widehat{t}}_{i}=\widehat{g}\left({t}_{i}\right)=\widehat{\alpha }+\widehat{\beta }{t}_{i}$ in the scale of log counts, or ${\widehat{T}}_{i}={2}^{\widehat{g}\left({t}_{i}\right)}-1={2}^{\widehat{\alpha }}{\left({T}_{i}+1\right)}^{\widehat{\beta }}-1$ in the scale of raw counts. If $\beta =1$ (single parameter form), ${\widehat{T}}_{i}={2}^{\widehat{\alpha }}{T}_{i}+\left({2}^{\widehat{\alpha }}-1\right)\approx {2}^{\widehat{\alpha }}{T}_{i}$, the normalization is almost a scaling. If $\beta$ is a free parameter (double parameter form), ${\widehat{T}}_{i}={2}^{\widehat{\alpha }}{\left({T}_{i}+1\right)}^{\widehat{\beta }}-1$, the resulting power law represents a simple nonlinear transform from ${T}_{i}$ to ${\tilde{T }}_{i}$ and vice versa. It means the scaling coefficients of the read counts of transcripts at different expression levels are allowed to be different. Thus, it has higher flexibility to model the possible non-uniformness in the steps of isolation, amplification, and sequencing of transcripts at low and high expression levels.

### Least trimmed squares regression

Now we consider the parameter estimation of the regression model (2). Given a constant integer $h, \frac{n}{2}<h<n$, the least trimmed squares (LTS) estimate of $\theta =\left(\alpha ,\beta \right)$ is defined as

$${\widehat{\theta }}^{\left(LTS\right)}=\underset{\theta }{\mathrm{argmin}}\sum_{i=1}^{h}{e}_{\left[i\right]}^{2}\left(\theta \right),$$

where ${e}_{\left[i\right]}^{2}\left(\theta \right)$ is the $i$-th order statistic of $\{{e}_{1}^{2}\left(\theta \right),\dots ,{e}_{n}^{2}\left(\theta \right)$}, where ${e}_{i}\left(\theta \right)={y}_{i}-\alpha -\beta {t}_{i}$.

The LTS estimate is regression, scale, and affine equivariant. The breakdown point of ${\widehat{\theta }}^{\left(LTS\right)}$ is roughly equal to the trimming proportion $(n-h)/n$. The LTS estimate can reach the maximal breakdown point $(((n-p)/2)+1)/n$ among the regression equivariant estimates when $h= [n/2]+ [(p+1)/2]$, where $[x]$ is the integer part of $x$ and $p=2$ in mode (2). Finally, it is $\sqrt{n}$-consistent and asymptotic normal in the case of continuously distributed disturbances [23].

At the value of the LTS estimate ${\widehat{\theta }}^{\left(LTS\right)}$, we sort the residuals by: ${e}_{[1]}^{2}\left({\widehat{\theta }}^{\left(LTS\right)}\right)\le {e}_{\left[2\right]}^{2}\left({\widehat{\theta }}^{\left(LTS\right)}\right)\le \dots {\le e}_{\left[h\right]}^{2}\left({\widehat{\theta }}^{\left(LTS\right)}\right)\le \dots {\le e}_{[n]}^{2}\left({\widehat{\theta }}^{\left(LTS\right)}\right)$, and empirically define the undifferentiated transcript set between a pair of reference and target samples as the transcripts corresponding to the smallest $h$ squares. Similar to the case of least squares, the following is true for LTS.

#### **Proposition 2**

*The trimmed average* $\frac{1}{n}\sum_{i=1}^{h}{e}_{\left[i\right]}({\widehat{\theta }}^{\left(LTS\right)})=0$.

That is, the average of the log ratios of the undifferentiated transcript set between a pair of samples is zero after normalization.

The above describe Part C of the MUREN workflow shown in Fig. 1. Next, we explain Part B.

### Selection of multiple reference samples

Suppose the RNA-seq samples are indexed by $\left\{\omega \in\Omega \right\}$. Denote the set of undifferentiated transcripts between two samples indexed by $\omega , \psi$ as ${\Lambda }_{\omega , \psi }$. Assume the criterion in the definition of undifferentiated transcripts sets remain the same across pairs of samples. The undifferentiated transcripts set of all the samples is given by $\Delta ={\bigcap }_{\omega , \psi \in\Omega }{\Lambda }_{\omega , \psi }$. As the size of $\Omega$ increases, $\Delta$ would be reduced, and possibly close to null. By the same token, the existence of housekeeping genes for a large collection of samples under a wide range of conditions is questionable. However, in such a situation, either housekeeping genes or undifferentiated transcripts between a pair of samples may still be defined.

There are some ways to select references. Biologically, we can select one or several samples under each experimental condition as references and align every target sample to the reference set. In this strategy, the experiment design of biology provides certain prior knowledge. Statistically, we can get hints from some exploratory data analysis. For examples, the hierarchical clustering arranges samples by some measure of distance/dissimilarity. Heuristically, we can select the samples on different branches as references. Last, it is straightforward to select all samples as references if sample size is relatively small, and select a random subset of samples as references if the sample size is large. In the examples shown in this report, slight differences were observed across different sets of references.

Next, we describe the model in Part D of the MUREN workflow as shown in Fig. 1.

### Transcript-wise integration of multiple pre-normalized counts

Suppose that a collection of $k$ samples are to be normalized. Among them, $l$ references are selected for pairwise normalization. Denote the pre-normalized count of ${t}_{i}$, the count in the $i$-th sample, with respect to the $j$-th reference by ${\widehat{t}}^{(ij)}={\widehat{\alpha }}_{ij}+{\widehat{\beta }}_{ij}{t}_{i}$, where ${\widehat{\alpha }}_{ij}$ and ${\widehat{\beta }}_{ij}$ are estimated in pairwise normalization. Suppose the target and reference effects are additive after log transformation, i.e.

3 $${\widehat{t}}^{(ij)}=\mu +{a}^{(i)}+{b}^{(j)}+{\epsilon }^{(ij)},$$

where $i=\mathrm{1,2},...,I, j=\mathrm{1,2},...,J$, $\mu , {a}^{(i)},{b}^{(j)},{\epsilon }^{(ij)}$ are the grand term, target effects, reference effects, and random errors respectively. We use this model to integrate the multiple pre-normalized counts into one final value by adjusting the reference effects. The final integrated log-count of the $i$-th sample is then $\widehat{\mu }+{\widehat{a}}^{(i)}$. We estimate the parameters in a robust way so as to avoid the unwanted influences caused by outlier reference samples (see Results). Different from the model of pairwise normalization, the model (3) is a two-factor model, whose design matrix is consisted of zeros and ones. This two-factor model (3) has a bounded design matrix. In this case, we choose to estimate the parameters by least absolute deviations (LAD) rather than least squares (LS).

To understand the rationale of the model (3), we consider the specific situation in which the scaling coefficients of the read counts from different samples are at the same level. Now it suffices to consider the single-parameter case where ${\beta }_{ij}$=1 and ${\alpha }_{ij}=0$. Since the LTS estimates in the pairwise normalization is consistent, that is, ${\widehat{\alpha }}_{ij}\approx 0$, both the sample and reference effects would be 0 approximately. In the original scale, the final scaling coefficients would be equal to 1 approximately. Namely, after normalization the counts would remain as they were.

### Least absolute deviations estimate and median polish

The model (3) is identifiable subject to the constrains: $\mathrm{median}\left\{{a}^{\left(i\right)}, i=1,\dots ,I\right\}=\mathrm{median}\left\{{b}^{\left(j\right)}, j=1,\dots ,J\right\}=0$. The LAD estimate of $\vartheta =(\mu ,{a}^{\left(1\right)},\dots ,{a}^{\left(I\right)},{b}^{\left(1\right)},\dots ,{b}^{(J)})$ is defined as

$${\widehat{\vartheta }}^{\left(LAD\right)}=\underset{\mu ,{a}^{\left(i\right)},{b}^{(j)}}{\mathrm{argmin}}\sum_{i=1}^{I}\sum_{j=1}^{J}\left|{\widehat{t}}^{(ij)}-\mu -{a}^{(i)}-{b}^{(j)}\right|.$$

Similar to the results in the three-factor model in [21], we can show that the LAD estimate is robust in the sense that the influence function of one observation is bounded. The influence function technically measures the effect of infinitesimal perturbation of one data point on the estimates. Not only is the LAD estimate robust, but also has some kind of efficacy. Its $\sqrt{n}$-consistence or asymptotic normality is valid under certain regularity conditions [24].

The general LAD can be formulated as a linear programming (LP) problem and thus be solved by the simplex or the interior point algorithm [25, 26]. For the specific two-factor model (3), we prefer a simpler method to compute the LAD estimates, namely, the median polish method proposed by Tukey [27].

### Efficient implementation of computation

In the integration step, one specific model of form (3) is assumed for each transcript, and the model parameters are not assumed to be related across transcripts. Consequently, the integration by median polishing is carried out for each transcript. However, in the single-parameter case where $\beta =1$, the integration can be simplified. Suppose in the pairwise normalization step, that the pre-normalized log counts of a specific transcript are ${\widehat{t}}^{(ij)}={t}_{i}+{\widehat{\alpha }}_{ij}$, where ${\widehat{\alpha }}_{ij}$ is the estimated parameter in the pairwise normalization of the $i$-th target with respect to the $j$-th reference. Plug it into model (3), we get

$${\widehat{t}}^{(ij)}={t}_{i}+{\widehat{\alpha }}_{ij}=\mu +{a}^{(i)}+{b}^{(j)}+{\epsilon }^{(ij)}$$

i.e.

$${\widehat{\alpha }}_{ij}=\mu +\left({a}^{(i)}-{t}_{i}\right)+{b}^{(j)}+{\epsilon }^{(ij)}.$$

The models of different transcripts become identical if we reparametrize ${a}^{(i)}$ by subtracting corresponding (log) counts ${t}_{i}$. Hence, the transcript-wise integration can be done through the integration of ${\widehat{\alpha }}_{ij}$’s. This is proved by the following proposition.

#### **Proposition 3**

(Once-for-all computation) *Consider the following two optimization problems,*

*M1:*

$$\begin{array}{c}\underset{\mu ,{a}^{\left(i\right)},{b}^{\left(j\right)}}{\mathrm{min}}\sum_{i=1}^{I}\sum_{j=1}^{J}\left|{t}_{i}+{\widehat{\alpha }}_{ij}-\mu -{a}^{\left(i\right)}-{b}^{\left(j\right)}\right|\\ \mathrm{s}.\mathrm{t}. \mathrm{median}\left\{{a}^{\left(i\right)}\right\}=\mathrm{median}\left\{{b}^{\left(j\right)}\right\}=0\end{array}$$

*M2:*

$$\begin{array}{c}\underset{\mu ,{a}^{\left(i\right)},{b}^{\left(j\right)}}{\mathrm{min}}\sum_{i=1}^{I}\sum_{j=1}^{J}\left|{\widehat{\alpha }}_{ij}-\mu -{a}^{\left(i\right)}-{b}^{\left(j\right)}\right|\\ \mathrm{s}.\mathrm{t}. \mathrm{median}\left\{{a}^{\left(i\right)}\right\}=\mathrm{median}\left\{{b}^{\left(j\right)}\right\}=0\end{array}$$

*If* ${\vartheta }_{2}=({\mu }_{2},{a}_{2}^{\left(1\right)},\dots ,{a}_{2}^{\left(I\right)},{b}_{2}^{\left(1\right)},\dots ,{b}_{2}^{\left(J\right)})$ *solves M2 then* ${\vartheta }_{1}={\vartheta }_{2}+({\mu }_{0},{t}_{1}-{\mu }_{0},\dots ,{t}_{I}-{\mu }_{0},0,\dots ,0)$ *solves M1, where* ${\mu }_{0}=\mathrm{median}\left\{{a}_{2}^{\left(i\right)}+{t}_{i}\right\}$.

The proof is essentially substitution of the corresponding variables. Then the integrated (log) count in the $i$-th sample is ${{\mu }_{2}+a}_{2}^{\left(i\right)}+{t}_{i}$, this holds for all transcripts. Moreover, in this case, the reference effects are the same across transcripts, which is indicated by the same ${b}^{\left(j\right)}$’s in ${\vartheta }_{1}$ and ${\vartheta }_{2}$. It implies that, even though in the general model (3) the parameters of the reference effects are not directly related across transcripts, they are identical in the single-parameter case. In other words, the adjustment of reference effect or the median polishing procedure only need to be carried out once for all the transcripts.

In the double parameter formulation, if we take LS estimate (${l}_{2}$-norm) instead of LAD estimate (${l}_{1}$-norm), replacing the constraints on medians by means, then model (3) is a two-factor ANOVA (analysis of variance) model with a complete design matrix. Consequently, the average (log) counts of the $i$-th sample are

$$\widehat{\mu }+{\widehat{a}}^{(i)}=\frac{1}{J}\sum_{j=1}^{J}\left({{\widehat{\alpha }}_{ij}+\widehat{\beta }}_{ij}{t}_{i}\right)={\overline{\alpha }}_{i}+{\overline{\beta }}_{i}{t}_{i},$$

where ${\overline{\alpha }}_{i}$ and ${\overline{\beta }}_{i}$ are the averages of ${\widehat{\alpha }}_{ij}$ and ${\widehat{\beta }}_{ij}$ over index $j$ respectively. Thus, the transcript-wise integration can be done through averaging coefficients ${\widehat{\alpha }}_{ij}$’s and ${\widehat{\beta }}_{ij}$’s for each transcript.

Unfortunately, the algebra of ${l}_{1}$-norm in LAD estimate is not so straightforward as that of ${l}_{2}$-norm in LS estimate. Heuristically, we can polish coefficients of slopes and intercepts respectively and apply the results to all transcripts. The fast alternative is competitive in computation time for large scale data.

## Results

### Dataset A

It (GSE47792 [28]) comes from the Sequencing Quality Control (SEQC) project [29]. The study contains five groups of experiments of rat toxicogenomics that produced 30 RNA-seq samples (n = 3). In each group the treated rats were fed or injected with one of the following drugs—methimazole (MET), 3-methylcholanthrene (3ME), betanapthoflavone (NAP), thioacetamide (THI), and *N*-nitrosodimethylamine (NIT); control rats were maintained without drugs. At the same time, all RNA samples were spiked in with the External RNA Controls Consortium [30] mixed sequences as the baseline truth. The ERCC sequences had four known control ratios of abundances, 1:1, 1:1.5, 1:2, and 4:1, respectively. Each ratio group consisted of 23 sequences distributed in a wide range of abundances.

Using dataset A we evaluate the accuracy of different methods by comparing their estimated ratios of ERCC sequences with the known control fold changes/ratios. Hereafter ratios and fold changes are used interchangeably. Then we show that the common methods are applicable to the cases of regular transcription profiles yet less effective in the cases of asymmetrically regulated transcription profiles (ART). In the latter, the patterns of up- and down-regulated transcripts between certain pair of samples are different. In statistical words, the expression differentiation is skewed. ART can be visualized by an asymmetric density plot and be summarized by the statistical measure—skewness of log ratios. We propose a guiding criterion of normalization: recover true (log) ratios while preserving the log ratios’ skewness due to its biological context.

### Dataset B

This [31] is from a plate-based single cell RNA-seq experiment of the murine multipotent myeloid progenitor cell line 416B transduced with oncogene CBFB-MYH11 (#cells = 192). The impact of log ratios’ skewness on normalization has been noticed in our past research [32], yet its biological meaning has not been addressed so far. Using dataset B we exemplify the skewness of log ratios biologically, thereby justify the above proposal. Specifically, we compare expressions of cells at different phases of cell cycles, and show that the differentiation between phases is indeed skewed.

### MUREN and other methods

MUREN has two available forms: the single-parameter (MUREN-sp) and the two-parameter (MUREN-dp). Other than MUREN, our evaluation and comparison also include Raw (Raw counts), CPM, Q (Quantile), RLE, RUV, TMM, TPM, and UQ (Upper Quartile [33]).

Notice that throughout the article, the log ratio (*M*-value) is defined as

$$\mathrm{logratio} = {\mathrm{log}}_{2}\left({\mathrm{Counts}}_{1}+1\right)-{\mathrm{log}}_{2}\left({\mathrm{Counts}}_{2}+1\right),$$

and the log average (*A*-value) is defined as

$$\mathrm{logaverage} =[{\mathrm{log}}_{2}\left({\mathrm{Counts}}_{1}+1\right)+{\mathrm{log}}_{2}\left({\mathrm{Counts}}_{2}+1\right)]/2.$$

### MUREN recovers the true expression ratios (Dataset A)

Because the abundance ratios of ERCC spike-in sequences are known, it is most persuasive to compare the ratios recovered by various methods with the corresponding nominal values.

**Fig. 2:**
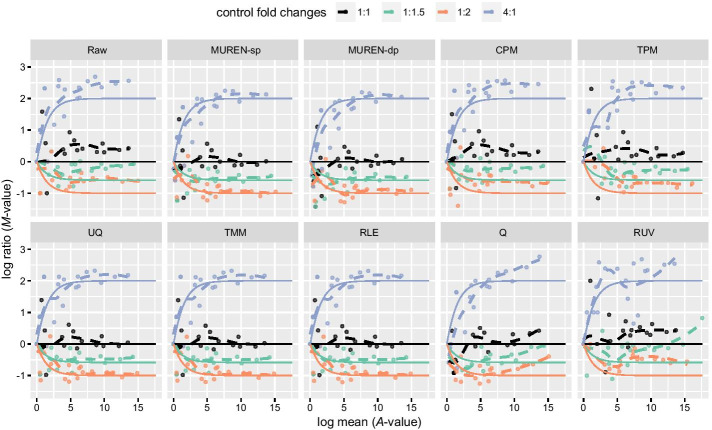
Comparison of the log ratios of the ERCC sequences using *M–A* plots obtained by different methods. RNA-seq data are from THI experiments. The x-axis represents averaged normalized read counts (*A*-value). The y-axis represents log ratios (*M*-value). The ERCC sequence groups of different preset ratios are shown in four colors. Dots: log ratio estimate of individual ERCC sequence after normalization; dashed lines: fitted values of the dots by local smoothing (LOWESS); solid lines: nominal relationships between *M*- and *A*-values. The results of MUREN, UQ, TMM, and RLE are fair; Those of CPM and TPM are inadequate; Those of Q and RUV are distorted

The results of THI experiments are illustrated by the enriched *M-A* plots in Fig. 2, in which the estimated log ratios are shown in points with fitted dashed lines, and the nominal values are shown in solid lines. Less difference indicates higher accuracy. Results of the unnormalized counts (method Raw) systematically deviate from the corresponding solid lines. The systematic bias indicates the necessity of normalization. The scaling methods, including MUREN-sp, UQ, TMM, RLE and the log-linear MUREN-dp, perform a fair normalization. In comparison, the methods CPM and TPM that assume constant total RNA contents or constant number of transcripts do not correct the counts adequately. In the opposite, the trends of estimated log ratios obtained by the nonlinear methods Q and RUV are heavily distorted. The similar results of other toxicogenomics experiments are shown in Additional file 1: Fig. S1–S4.

### MUREN preserves the asymmetrically regulated transcriptome (Dataset A)

**Fig. 3:**
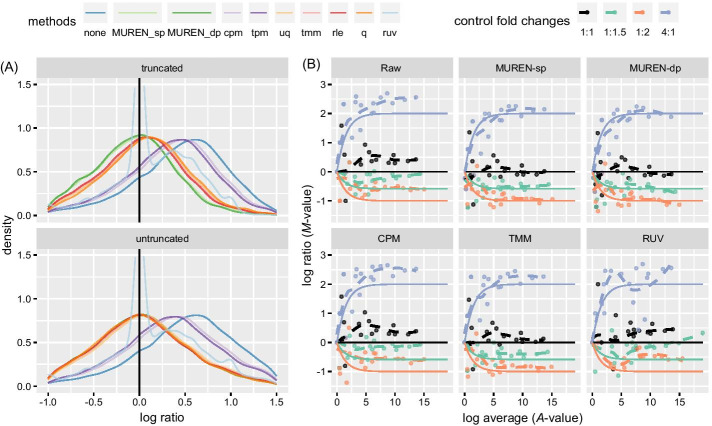
Evaluation of different normalization methods by the truncated transcriptome in THI experiments. **A** Top panel: densities of log ratios of the truncated transcriptome. After truncation, RLE, UQ, TMM, and Q are disturbed by the truncation/asymmetry; the modes of log ratios’ densities from these methods shift notably towards right. MUREN is resistant against the disturbance and keeps its mode near zero. Bottom panel: densities of log ratios of the untruncated transcriptome. The methods CPM and TPM cannot adjust the modes of densities near zero. **B** *M–A* plots of ERCC sequences of selected methods applied to the truncated transcriptome. Compared with Fig. 2, TMM has larger deviations between the estimated and expected log ratios due to the influence of truncation. It cannot normalize the counts as well as it does for the untruncated transcriptome. By comparison, MUREN is resistant against the asymmetric perturbation. See Additional file 1: Fig. S5 for the results of other methods

When the transcriptomic differentiation profile is (nearly) symmetric, namely the distribution of transcript-wise log ratios is (nearly) symmetric, the up- and down-regulated transcripts are comparable. In this case, we cannot see much difference between MUREN, TMM, RLE, and UQ as shown in Fig. 2. The bottom panel in Fig. 3a shows the densities of normalized log ratios of all transcripts in THI experiments. The modes of the densities of most methods are near zero, except CPM, TPM, and Raw. The near-zero mode is an indicator of appropriate normalization, and this point will be elaborated later.

In the following evaluation, we perturbed the THI data by truncating the transcriptome at the right tail as follows: first, summarize the counts by the medians of the three replicates respectively for the control and treatment samples; second, sort the transcripts in the ascending order by the ratios of the summarized treatment and control counts; finally, remove the top 15% transcripts from all samples. The truncated transcriptome is more asymmetric than the original one is.

As shown in Fig. 3a (top panel), the asymmetry of the transcriptome results in the densities of RLE, UQ, TMM, and Q (orange and red lines) shifting towards right, which produces systematic biases. These methods are disturbed by the introduction of asymmetry. By contrast, MUREN (green lines) is more resistant against the asymmetric perturbation and keeps its mode around zero. Notice that the results of CPM and TPM (violet lines) and Raw (blue line) leave their modes far away from zero. RUV (light blue line) has a sharp peak around zero which is trimmed by the *y*-axis limit. Even though the mode of RUV is not influenced, the shape of log ratios’ density is distinctively changed. See Additional file 1: Fig. S5 for a zoomed scope of densities.

Back to the ERCC sequences, Fig. 3b shows the *M–A* plots of ERCC sequences with selected methods applied to the truncated transcriptome. The results coincide with those in Fig. 3a. Compared with Fig. 2, we see obvious deviations, disturbed by the truncation, between the fitted (dashed) lines and corresponding theoretical (solid) lines in the result of TMM. At the same time MUREN is immune to the asymmetry. See Additional file 1: Fig. S6 for the results of other methods.

### Evaluate goodness of normalization by the densities of log ratios (Dataset A)

Part E concerns the evaluation of normalization. According to Proposition 3, the trimmed average of the log ratios between a pair of samples is zero after pairwise normalization. If the log ratios of the undifferentiated transcripts set are roughly symmetric, then the mode of log ratios' density would be near the trimmed average, which is zero. This assumption is reasonable because the differentiations of housekeeping genes should be due to random fluctuations. Since we impose the restriction that median of the reference effect in (3) is zero, the mode would be near zero too after integration, Conversely, if the mode is near zero, it implies that the expressions of a majority of transcripts remain unchanged. Shown at the bottom in Fig. 3a are examples of the log-ratio densities of THI experiments. The log ratios of unnormalized counts show a unimodal distribution. After normalization, the mode is shifted near zero in all cases except for the results of CPM and TPM.

Another informative feature of the density is its shape. Later we will offer, by a typical example, a biological interpretation of the skewness of transcriptomic differentiation. Thus, we recommend the normalization should not change the overall skewness or modality of log ratios’ distribution. Too flexible methods, usually nonlinear methods, tend to change the shape. The log ratios’ density together with the *M–A* plot offers a rather comprehensive diagnosis of the normalization goodness.

### MUREN preserves the skewness of log ratios (Dataset A)

The shape of log ratios’ distribution, characterized by such as unimodality/multimodality and skewness, is a biological signature of transcriptomic differentiation. We propose that the aim of normalization has two folds: first, improve the accuracy of the log ratios; second, preserve the overall shape of log ratios’ density. Normalization, for example, should neither turn a positively skewed distribution to a negatively distributed one, nor turn a unimodal distribution to a multimodal one.

**Fig. 4:**
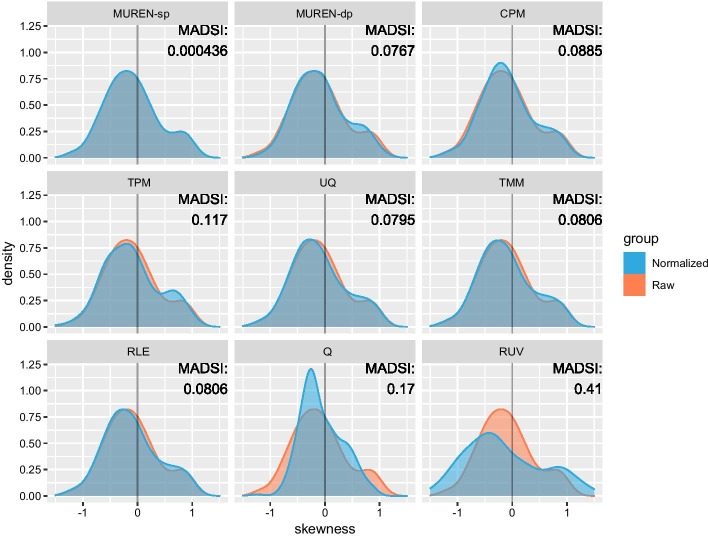
The distributions of pairwise skewness. For every pair from the pooled samples of the five rat toxicogenomics experiments, the skewness of log ratios is computed. Orange: skewness distribution of raw counts; blue: skewness distribution of normalized counts. The divergence between them is measured by the mean absolute deviations index of skewness (MADSI). The linear methods do not change the skewness too much; among them, MUREN-sp has the minimum value of MADSI. However, the nonlinear methods like Q and especially RUV tend to alter the skewness boldly

Hereafter, we quantify the skewness by the empirical measure $S=\frac{1}{n} {\sum }_{i=1}^{n}{\left(\frac{{x}_{i}-\mu }{\sigma }\right)}^{3}$, where $\mu$ is the sample mean and $\sigma$ is the sample standard deviation. For each pair among the pooled samples from the five groups of the rat toxicogenomics experiments, we compute the pairwise skewness. Next, we consider the collection of all pairwise skewness for raw counts and normalized counts, and denote them respectively by ${\{S}_{i}\}$ and $\{{S}_{i}^{^{\prime}}\}$. To measure the overall skewness difference between them, we define the mean absolute deviations index of skewness (MADSI) as $\mathrm{MADSI}=\frac{1}{m}{\sum }_{i=1}^{m}|{S}_{i}-{S}_{i}^{^{\prime}}|$. Smaller MADSI indicates smaller change of skewness. The results are shown in Fig. 4. As we can see, the linear methods do not change the skewness too much, among them MUREN-sp has the minimum value of MADSI. However, the nonlinear methods like Q and especially RUV tend to alter the skewness boldly.

### Normalization with multiple references is more reliable than that with a single one (Dataset A)

We have explained the necessity of normalization with multiple references in the theoretical setting. In practice, the results using single reference may not have much difference with those using multiple references, provided the differentiation is relatively small and the data quality is high. But we cannot rule out the possibility that an outlier sample in the dataset due to contamination or errors in the sequencing process would be taken as the reference. Normalization with multiple references is not influenced by individual outlier reference sample while the normalization with a single reference is influenced severely. Indeed, this is confirmed by simulations in which some samples were artificially disturbed by increasing or decreasing the counts randomly, see Additional file 1: Fig. S7 for details.

### Skewed transcriptomic differentiation corresponds to increased/decreased activities of cells (Dataset B)

When we compare transcriptomes of two samples, positive/negative skewness of log ratios are characterized by a heavy right/left tail. This implies that certain biological processes are significantly up- or down-regulated from one sample to the other, Next, we show such an example of cell cycle transitions using single cell RNA-seq data.

Dataset B is from a single cell RNA-seq experiment spanning over different cell cycle phases: G1, S, G2, and M. G1 is the first growth phase, and rates of RNA transcription and protein synthesis are high; S is the DNA replication phase, in which most other biosynthesis turns lower; G2 is the growth phase preparing the cell for mitosis; the relatively short M phase undergoes cell division. We normalize the counts of each cell with its total counts. Using the tool implemented in the R package scran [34, 35] to annotate the cell cycle, we identify 50, 19, and 35 cells in the G1, S, and G2/M phases respectively with normalized cell cycle scores > 0.6.

**Fig. 5:**
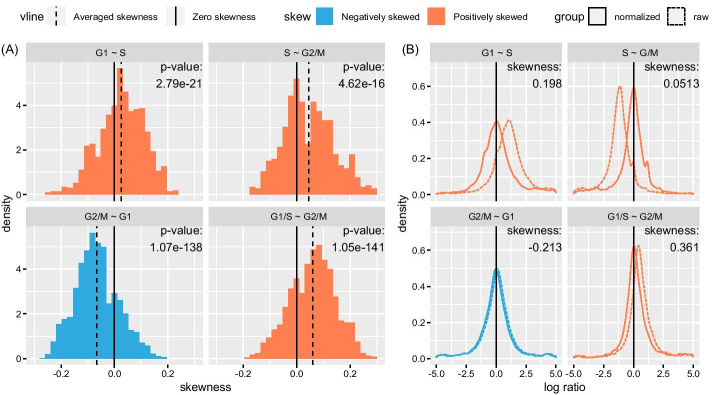
Cell cycle scRNA-seq data: the transition of phases is associated with the skewness of log ratios at both the single cell and the bulk level. **A**The histograms of pairwise skewness of log ratios between cells at different phases. Left top: compared with cell in S phase, the cells in G1 phase are more active, in brief notation, G1 > S, and the transcriptomic differentiation is positively skewed; Right top: S > G2/M; left bottom: G2/M < G1; right bottom: G1/S > G2/M. Under the null hypothesis that the skewness is randomly positive or negative with equal probabilities, the nonparametric sign tests report extremely significant *p*-values. **B** Log ratios’ densities of pseudo bulk counts obtained by summing over cells in the same phase. After normalization by MUREN-sp, the modes of the densities of the pseudo bulk counts align to zero. The skewness of (normalized) bulk log ratios is consistent with that at the single cell level. Notice that the skewness of normalized and unnormalized log ratios has little difference

If we compare the expression profiles of G1 versus S phases, the distribution of the log ratios is expected to be positively skewed because general biological processes involved in growth are more active in G1 phase than in S phase. Similarly, the distribution between S and G2/M phases is expected to be positively skewed; the distribution between G2/M and G1 phases is expected to be negatively skewed. Indeed, as shown in Fig. 5a, the distributions of pairwise skewness between cells at different phases validate the above conjectures. Moreover, under the null hypothesis that the skewness is randomly positive or negative with equal probabilities, the nonparametric sign tests report extremely significant *p*-values. The conclusions agree with the changes of activities along the cell cycle phases.

### Enlarged skewness of log ratios in pseudo-bulk transcriptomes (Dataset B)

To investigate skewness at the bulk level, we merge the counts of cells in the same phase into pseudo bulk RNA-seq counts and normalize them with MUREN-sp. The diagnostic density plots along with the skewness are shown in Fig. 5b, in which MUREN adequately normalizes the pseudo bulk RNA-seq counts. The meaning of log ratios’ density is the same as what we interpret in the above. Moreover, the skewness of transcriptomic differentiation at the pseudo bulk level is not only consistent with that at the single cell level, but also enlarged. Take G1/S ~ G2/M for example, the cell level skewness is overall positive (see Fig. 5a), yet none of the pairwise skewness exceeds 0.3. However, the skewness of pseudo bulk counts reaches as large as 0.376 (Fig. 5b), which is larger than the maximal skewness at the cell level. The same conclusion is true in the other three comparisons. Thus, using this single cell RNA-seq data, we exemplified the skewness of biological differentiation at both the single cell and the bulk level.

## Discussion

In this report, we address the issue—goodness of normalization in two aspects: (1) improve the accuracy of normalization; (2) preserve the skewness of differentiation. Specifically, we check the density plots of expression differentiation along with the *M–A* plots. The mode and skewness of the density are important indicators of normalization goodness.

The undifferentiated transcripts set between a pair of samples is consistent with the notion of housekeeping genes. With appropriate normalization, as we have shown, the average of the log ratios of the undifferentiated transcripts set is zero. Compared with trimmed average, mode can be visualized for diagnosis. If the symmetric assumption about the undifferentiated transcripts set approximately holds, then the mode of pairwise expression differentiation should be near zero, see Fig. 3a for such cases. Otherwise, as the mode shifts seriously away from zero, the differentiation of all other genes will be biased, and the quantification of up- and down-regulation would be biased, see the cases of inappropriate normalization in Figs. 2 and 3b. The unbiased quantification of gene differentiation is crucial for downstream analysis such as gene set enrichment [36, 37], low rank decomposition [38], and inference of transcriptional regulation [39, 40]. Unbiased quantification of differentiation is the basis of DE gene calling as well. R packages such as edgeR [12, 15] and DESeq2 [13] model the raw counts by the negative binomial (NB) distribution with covariates, to call DE genes. The scaling factor estimated by MUREN-sp can be used to substitute the library size factor in edgeR and DESeq2 as an alternative, especially in the asymmetrically regulated transcriptome.

The ability of preserving the asymmetrical differentiation or skewness of data varies across different normalization methods as shown in the examples in Fig. 4. In particular, MUREN preserves the skewness using LTS, which has a breakdown value as high as 50%. According to the definition of breakdown value, the portion of data that deviates from the principal component could be of any kind pattern including skewness [41]. Such examples can be found in [42].

This proposed approach does not depend on a parametric model models such as Poisson distributions or negative-binomial distributions on the read counts. The method is applicable to any dataset as long as the assumption that more than 50% genes are both undifferentiated and are not subject to distortion between a sample and a reference is valid.

The MUREN implemented in R package is ready for daily normalization of RNA-seq data. MUREN has an efficient implementation and is integrated with a parallel R package. For the THI data (6 samples), it takes less than half a minute with single thread on a generic desktop computer. For large datasets, the parallel implementation can be specified by one line of code.

At the beginning of normalization, we log-transform the raw counts plus an offset *c*, see Fig. 1a. We recommend the offset to be 1 for two main reasons. First, the raw counts are nonnegative, and the log-transformed counts are also nonnegative. Moreover, log_2_(0 + 1) = 0, which means zero observed count is still zero after transformation. Second, the fold change of low counts is vulnerable and radical. The offset 1, indeed, shrinks the fold change to zero. Consider two raw counts 4 and 0, the fold change is infinite which is unreliable. Actually, we cannot determine the fold change accurately in this situation. Hence, a shrinkage of the fold change to zero is reasonable. When the offset is 1, log_2_(4 + 1) − log_2_(0 + 1) = 2.3; when the offset is 0.0001, log_2_(4 + 0.0001) − log_2_(0 + 0.0001) = 15.3.

## Conclusions

MUREN performs the RNA-seq normalization using a two-step statistical regression induced from a general principle. We propose that the densities of pairwise differentiations are used to evaluate the goodness of normalization. MUREN adjusts the mode of differentiation toward zero while preserves the skewness due to biological asymmetric differentiation. Moreover, by robustly integrating pre-normalized counts with respect to multiple references, MUREN is immune to outlier samples.

## Supplementary Information

**Additional file 1**. Supplementary_file.pdf.

## Data Availability

The RNA-Seq and scRNA-Seq datasets we used for comparison and/or as evidence are from GSE47792 [28] and [31].

## Abbreviations

3ME: 3-Methylcholanthrene
A-value: Log average
ART: Asymmetrically regulated transcription profiles
CPM: Counts Per Million
DE: Differentially expressed
ERCC: External RNA Controls Consortium
FPKM: Fragments per Kilobase per Million mapped reads
LAD: Least absolute deviations
LTS: Least trimmed squares
M-value: Log ratio
MADSI: Mean absolute deviations index of skewness
MET: Methimazole
MUREN: Multiple-reference normalizer
NAP: Betanapthoflavone
NIT: *N*-Nitrosodimethylamine
Q: Quantile method
RLE: Relative Log Expression
RNA-seq: RNA sequencing
RPKM: Reads per Kilobase per Million mapped reads
RUV: Remove Unwanted Variation
THI: Thioacetamide
TMM: Trimmed Means of M-values
TPM: Transcripts Per Million
UQ: Upper Quartile
