# Supplementary material for: MUREN: a robust and multi-reference approach of RNA-seq transcript normalization
Source: BMC Bioinformatics. 2021 Jul 28;22:386. doi: 10.1186/s12859-021-04288-0 (PMC8317383; doi:10.1186/s12859-021-04288-0)
Supplement: Supplementary file 1 — Additional file 1. Supplementary_file.pdf. [file 12859_2021_4288_MOESM1_ESM.pdf]

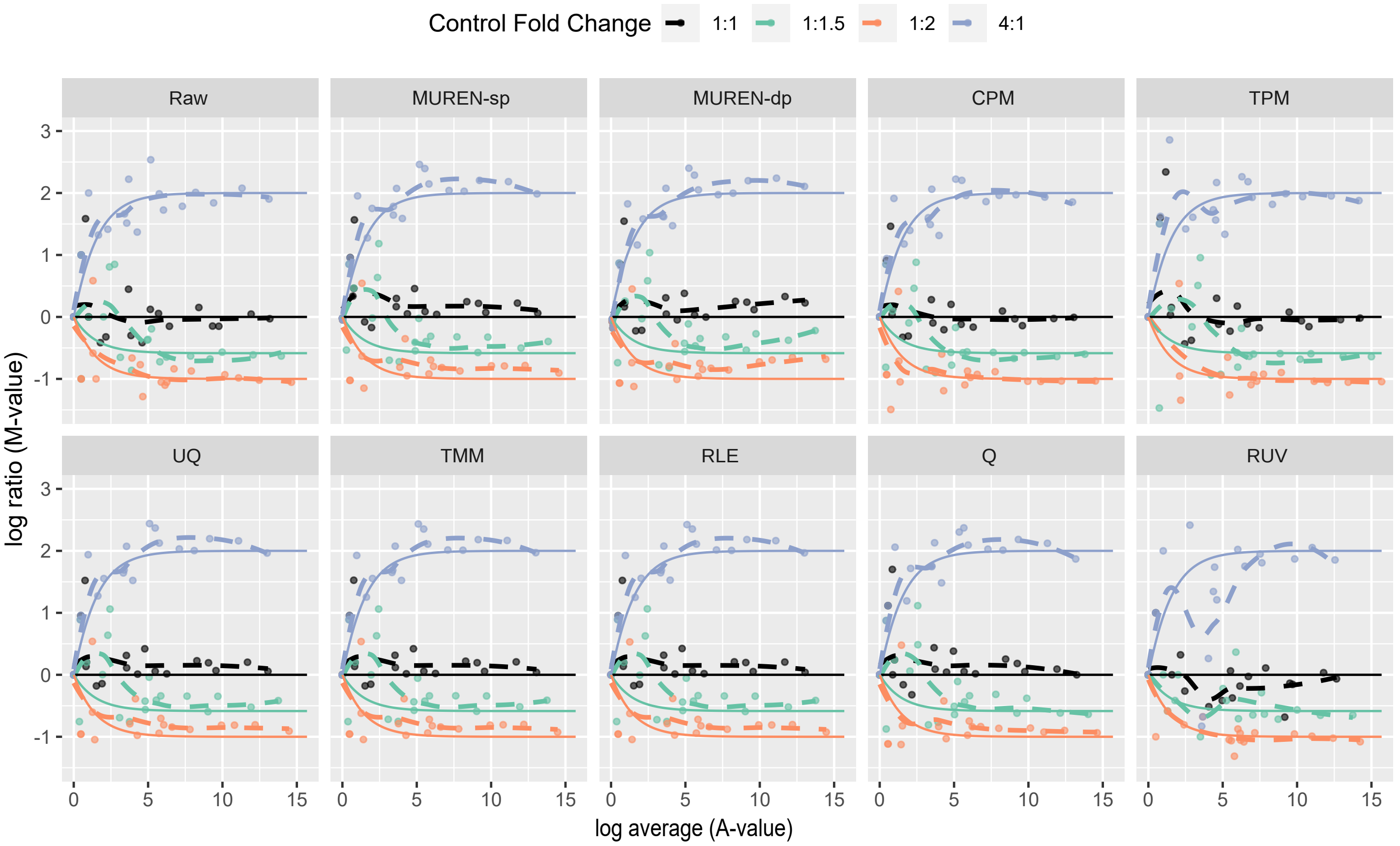

Figure S1: **3ME group: comparison of the log ratios of the ERCC sequences obtained by different methods.** The x-axis represents averaged normalized read counts (A-value). The y-axis represents log ratios (M-value). The ERCC sequence groups of different preset ratios are shown in four colors. Dots: log ratio estimate of individual ERCC sequence after normalization; dashed lines: local smooth fitting (LOWESS) of dots; solid lines: nominal relationship between M- and A-values.

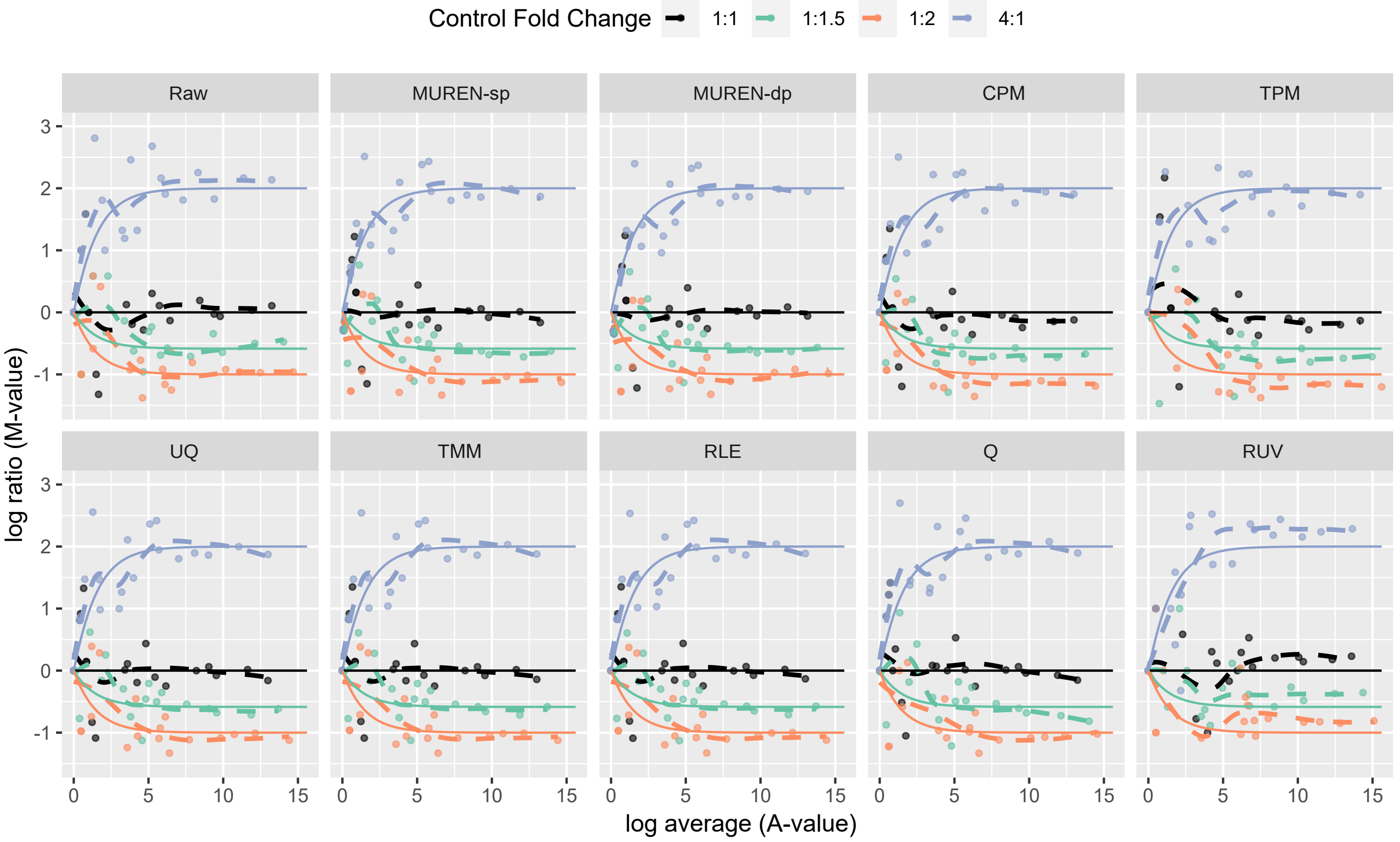

Figure S2: **MET group: comparison of the log ratios of the ERCC sequences obtained by different methods.** The x-axis represents averaged normalized read counts (A-value). The y-axis represents log ratios (M-value). The ERCC sequence groups of different preset ratios are shown in four colors. Dots: log ratio estimate of individual ERCC sequence after normalization; dashed lines: local smooth fitting (LOWESS) of dots; solid lines: nominal relationship between M- and A-values.

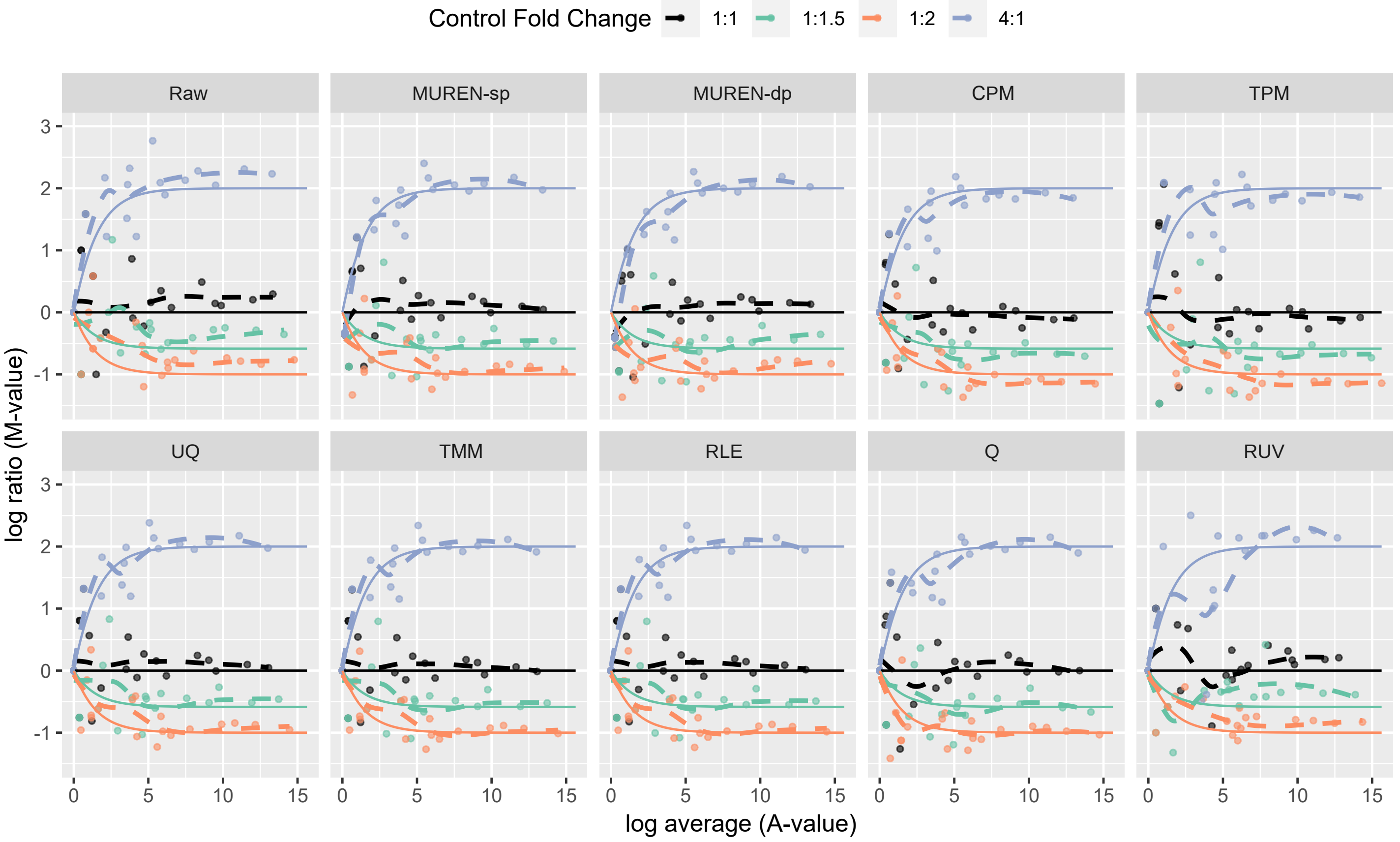

Figure S3: **NAP group: comparison of the log ratios of the ERCC sequences obtained by different methods.** The x-axis represents averaged normalized read counts (A-value). The y-axis represents log ratios (M-value). The ERCC sequence groups of different preset ratios are shown in four colors. Dots: log ratio estimate of individual ERCC sequence after normalization; dashed lines: local smooth fitting (LOWESS) of dots; solid lines: nominal relationship between M- and A-values.

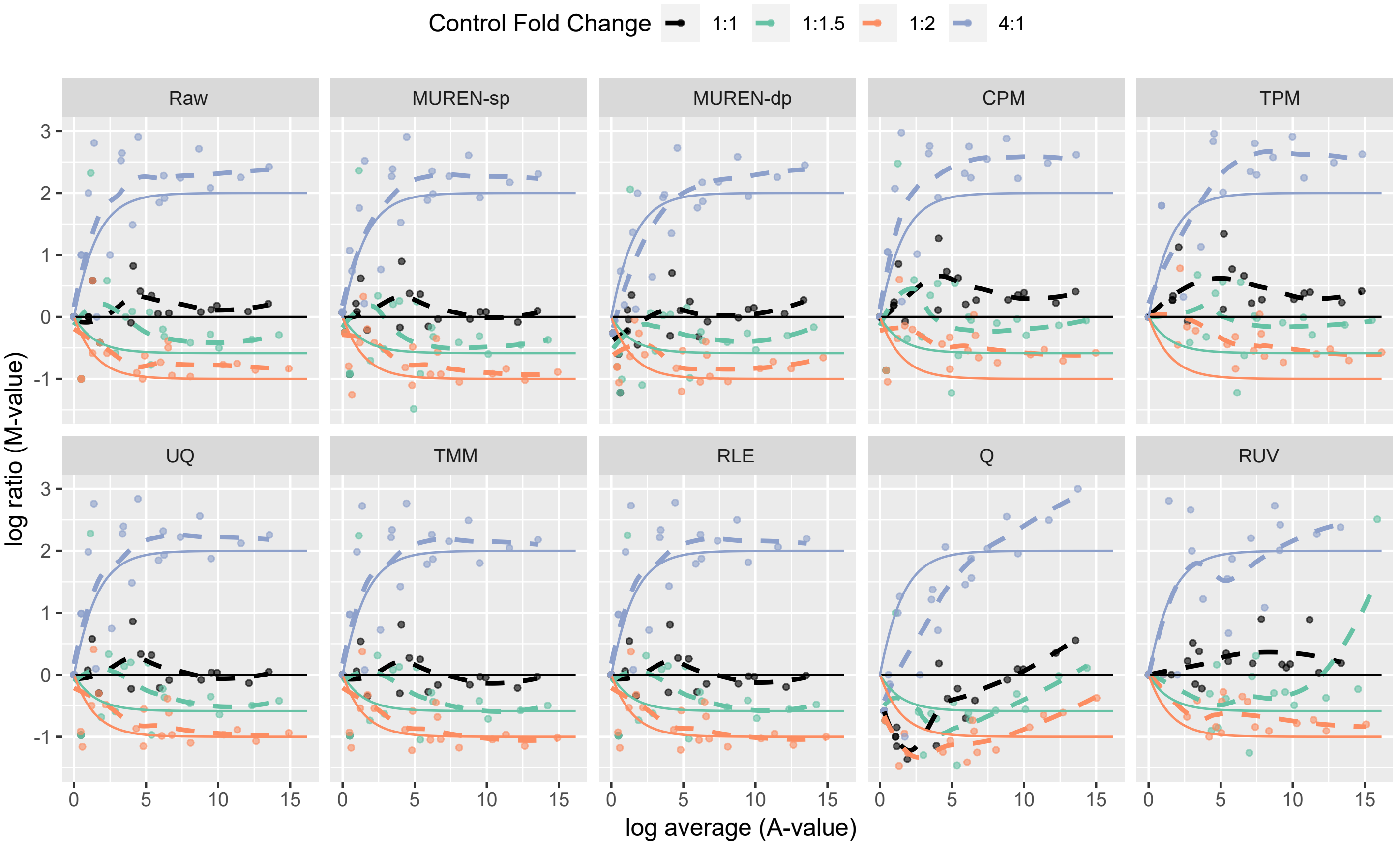

Figure S4: **NIT group: comparison of the log ratios of the ERCC sequences obtained by different methods.** The x-axis represents averaged normalized read counts (A-value). The y-axis represents log ratios (M-value). The ERCC sequence groups of different preset ratios are shown in four colors. Dots: log ratio estimate of individual ERCC sequence after normalization; dashed lines: local smooth fitting (LOWESS) of dots; solid lines: nominal relationship between M- and A-values.

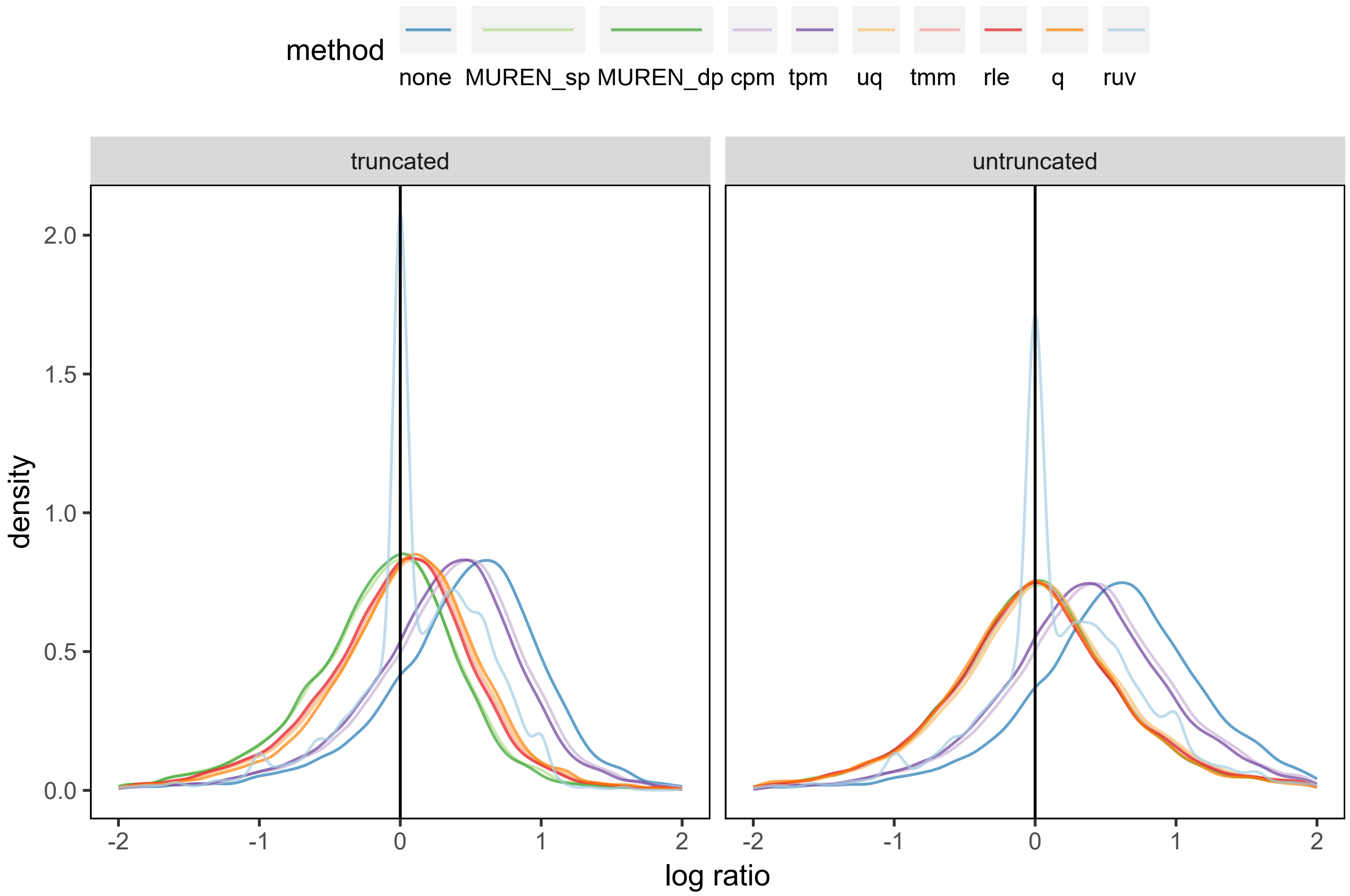

Figure S5: **Log ratios' densities of all transcripts with different methods applied to the truncated and untruncated transcriptomes in THI experiments.**

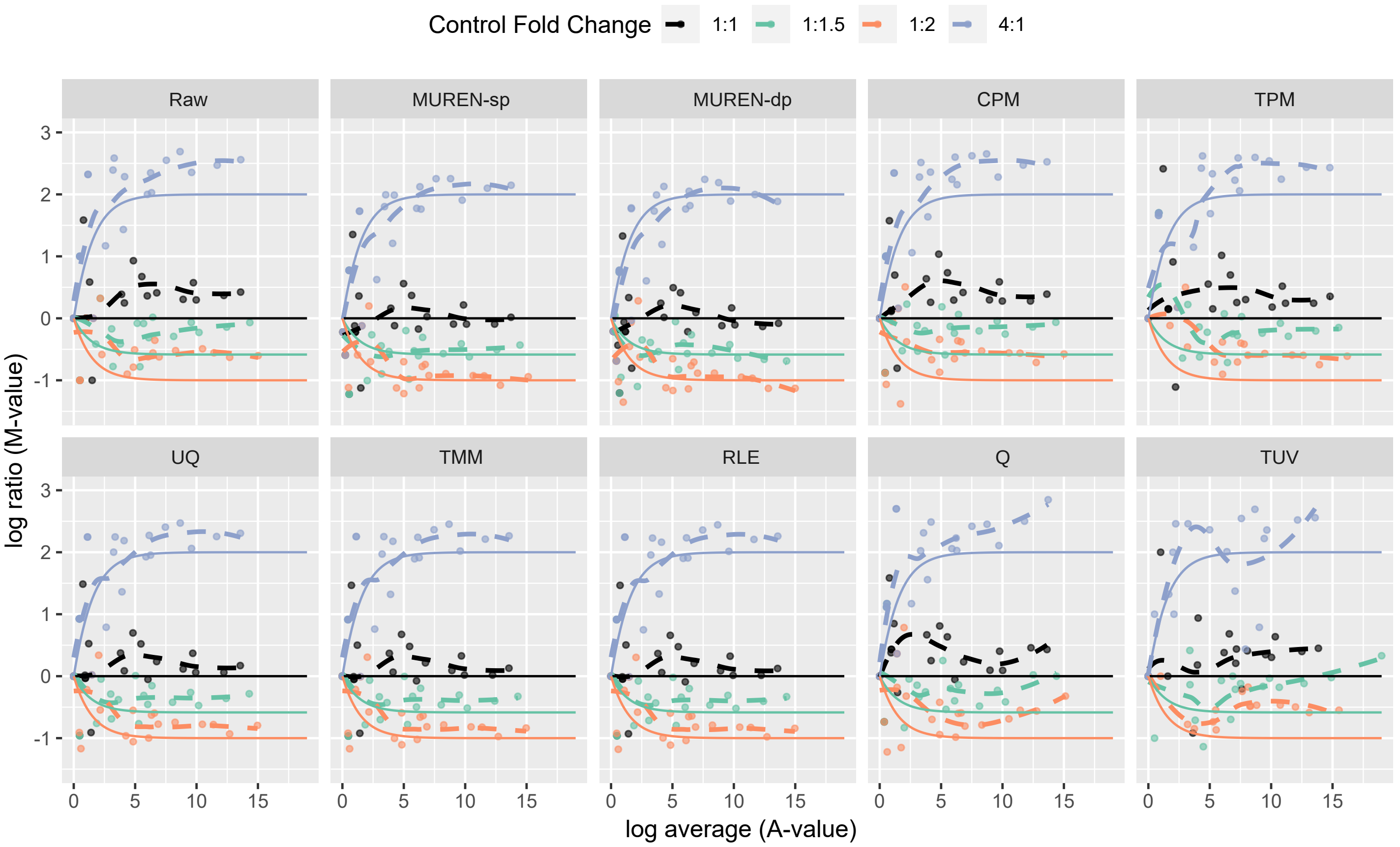

Figure S6: **M − A plots of ERCC sequences of different methods applied to the truncated transcriptome in THI experiments**

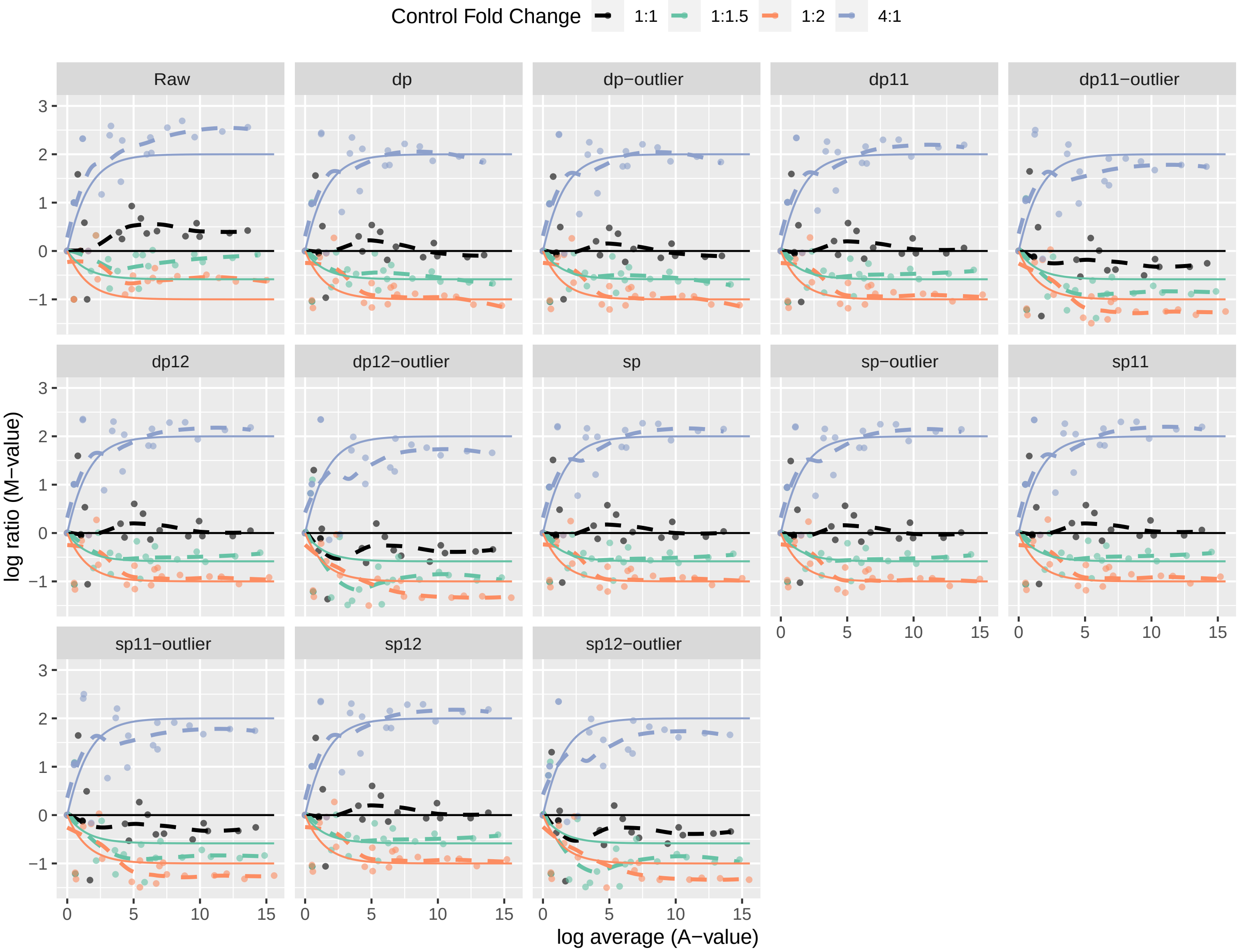

Figure S7: **Comparison of MUREN with single and multiple references.** dp(sp): MUREN-dp(sp) with all samples as references; dp(sp)11(12): MUREN-dp/sp with 11(12)-th samples as reference; dp(sp)11(12)-outlier: MUREN-dp/sp with outlier 11(12)-th samples as reference. The outlier samples are generated by increasing or decreasing the counts by 50% randomly. It's terrible to select the outlier sample as reference. By applying multiple references, we could avoid the unfortunate case. MUREN with multiple references is not influenced by individual outlier reference sample.
